# Advancements in Therapeutic Approaches for Proliferative Vitreoretinopathy: A Comprehensive Review

**DOI:** 10.7759/cureus.65893

**Published:** 2024-07-31

**Authors:** Kasturi K Dhawale, Pravin Tidake

**Affiliations:** 1 Ophthalmology, Jawaharlal Nehru Medical College, Datta Meghe Institute of Higher Education and Research, Wardha, IND

**Keywords:** gene therapy, biological therapies, pharmacological interventions, vitrectomy techniques, retinal detachment, proliferative vitreoretinopathy (pvr)

## Abstract

Proliferative vitreoretinopathy (PVR) is a significant complication of retinal detachment surgery, characterized by the growth of fibrous membranes that can lead to recurrent retinal detachment and vision loss. This comprehensive review aims to summarize the latest advancements in the therapeutic approaches for PVR, encompassing historical perspectives, current surgical techniques, pharmacological interventions, biological and genetic therapies, and novel experimental treatments. Traditional surgical methods, such as vitrectomy, have been refined with advanced instrumentation and techniques to improve outcomes. Pharmacological treatments, including anti-inflammatory and anti-proliferative agents, are being explored to prevent and manage PVR. Emerging therapies, such as stem cell and gene therapy, offer promising new avenues for treatment. Despite these advancements, challenges remain in preventing recurrence and improving long-term outcomes. This review highlights the progress made and identifies areas for future research, emphasizing the importance of continued innovation to enhance patient care and reduce the burden of PVR.

## Introduction and background

Proliferative vitreoretinopathy (PVR) is a complex pathological condition characterized by the formation of fibrous membranes within the retina and vitreous body, leading to retinal detachment and severe visual impairment. It is often considered the most common cause of failure in retinal detachment surgeries [1]. The pathophysiology of PVR involves a cascade of cellular and molecular events, including the proliferation and migration of retinal pigment epithelial (RPE) cells, glial cells, and fibroblasts into the vitreous cavity. These cells contribute to membrane formation and subsequent contraction, resulting in tractional retinal detachment. Inflammatory cytokines, growth factors, and extracellular matrix components play crucial roles in the development and progression of PVR [2]. PVR occurs in approximately 5-10% of patients undergoing primary retinal detachment surgery, making it a significant clinical concern. The incidence of PVR increases in cases of giant retinal tears, penetrating ocular trauma, and severe intraocular inflammation. Risk factors for developing PVR include the extent and duration of retinal detachment, the presence of vitreous hemorrhage, previous retinal detachment surgeries, and certain genetic predispositions [3]. Additionally, systemic conditions such as diabetes mellitus and hypertension have been associated with an increased risk of PVR. Understanding these risk factors is essential for identifying patients at higher risk and tailoring preventative strategies accordingly [4].

Research into PVR is crucial due to its impact on visual outcomes and quality of life for affected patients. Advances in the understanding of the molecular and cellular mechanisms underlying PVR have led to the development of novel therapeutic strategies aimed at preventing and managing this condition [5]. Improved surgical techniques, pharmacological interventions, and biological therapies have shown promise in reducing the incidence and severity of PVR. Ongoing research efforts are focused on identifying biomarkers for early detection, understanding the genetic basis of susceptibility, and exploring innovative treatments to enhance the efficacy of current approaches. The ultimate goal is to improve patient outcomes and reduce the burden of this debilitating condition [6]. This review aims to provide a comprehensive overview of the advancements in therapeutic approaches for PVR. This review will cover historical perspectives, current surgical techniques, pharmacological interventions, biological and genetic therapies, novel experimental treatments, and the challenges and future directions in PVR management. By synthesizing the latest research findings and clinical data, this review aims to highlight the progress made in the field and identify areas where further investigation is needed. This information will be valuable for clinicians, researchers, and stakeholders involved in the care and management of patients with PVR.

## Review

Historical perspective

Early Understanding and Initial Treatments

In the early days, PVR was referred to as “massive vitreous retraction” and “massive periretinal proliferation.” The term “proliferative vitreoretinopathy” was coined in 1989 by the Silicone Oil Study group to describe the disease that develops as a complication of rhegmatogenous retinal detachment (RRD) [7]. Initially, PVR was believed to be a cytokine-driven process in the vitreous, facilitating the abnormal proliferation of RPE cells. It was characterized by the development of retinal stiffness and contractile membranes on the surface or underside of the retina [8]. The primary treatment approach for PVR was surgical, focusing on reattaching the detached retina. However, the visual outcomes of these surgeries were very poor. Predisposing factors for PVR, such as preoperative PVR, aphakia, high levels of vitreous proteins, the duration of retinal detachment before corrective surgery, the size of the retinal hole or tear, intraocular inflammation, vitreous hemorrhage, and trauma to the eye, were identified [2]. Over time, researchers began exploring various adjunctive agents for preventing and treating PVR, such as corticosteroids, mitomycin C, bevacizumab, and ranibizumab. Despite promising preclinical results, many agents failed to demonstrate consistent benefits in human trials [1].

Evolution of Surgical Techniques

The evolution of surgical techniques has been a remarkable journey, marked by significant advancements that have transformed the field of surgery over the centuries. From ancient practices rooted in trial and error to the precision of contemporary minimally invasive procedures, surgical techniques have constantly evolved to improve patient outcomes, reduce complications, and enhance recovery times [9]. Early surgical practices in ancient civilizations such as Egypt, Greece, and Rome were characterized by crude instruments and a limited understanding of anatomy, making procedures risky. The Middle Ages witnessed slow progress, but the Renaissance marked a turning point with the anatomical studies of figures such as Leonardo da Vinci and Andreas Vesalius. The 19th century saw the emergence of anesthesia and antiseptic techniques, pioneered by figures such as Joseph Lister, enabling more complex surgeries with reduced risk [10].

The 20th century brought an explosion of technological innovations that reshaped surgery, including the development of X-ray technology for better preoperative planning and postoperative assessment and the discovery of antibiotics to reduce the risk of infections. The latter half of the century saw the rise of minimally invasive techniques, also known as laparoscopic or keyhole surgery, marking a significant milestone in surgical advancement by reducing surgical trauma and enabling faster recovery times [9]. Robotic surgery has emerged as a transformative technology, allowing for greater precision, flexibility, and control during complex procedures. Robotic platforms such as the da Vinci Surgical System have been widely adopted across various surgical specialties, offering enhanced visualization, improved dexterity, and reduced surgeon fatigue. The evolution of robotic surgery has been driven by advancements in computer science, engineering, and materials science, leading to the development of more sophisticated and user-friendly systems [11].

Artificial intelligence (AI) is poised to revolutionize surgical practice by reducing variability and enhancing patient outcomes. AI-powered tools can analyze surgical videos to generate valuable insights, helping to define and disseminate best practices worldwide. Routinely capturing and structuring intraoperative video data and patient characteristics and outcomes can provide valuable insights into surgical best practices and drive continuous improvement [12]. The evolution of surgical techniques has been a remarkable journey, with each advancement building upon the knowledge and innovations of the past. As technology continues to advance and interdisciplinary collaboration expands, the future of surgery appears promising, holding the potential to improve patient outcomes further and redefine the boundaries of medical possibility [13].

Limitations of Traditional Approaches

Despite advancements in surgical techniques, traditional approaches to treating PVR have several limitations. Even with improved surgical methods, the anatomical and visual outcomes in patients with PVR remain unsatisfactory. PVR remains the leading cause of failure in retinal detachment surgery and poses a significant challenge for vitreoretinal surgeons [14]. Surgeons may unknowingly leave residual vitreous layers during surgery, contributing to the development of PVR despite the use of advanced techniques. Incomplete vitreous removal can serve as a scaffold for cellular proliferation and contraction. Various pharmacological agents, such as corticosteroids, have been evaluated as potential preventive therapies for PVR, but clinical trials have yielded variable and disappointing results. Despite promising preclinical data, many agents have failed to demonstrate consistent benefits in human trials [2]. While our understanding of PVR pathophysiology has grown based on cellular and animal models, key aspects of the disease process remain incompletely understood. This limits the development of targeted therapeutic strategies. The lack of a standardized surgical approach makes evaluating the efficacy of adjunctive pharmacological interventions challenging. Proper patient selection and surgical technique standardization are crucial for assessing the impact of novel therapies [15].

Current surgical approaches

Vitrectomy Techniques

A vitrectomy is a surgical procedure to remove some or all of the vitreous humor from the eye. Various techniques are used in vitrectomy surgery, each tailored to address specific ocular conditions and complications. Pars plana vitrectomy is the most common type of vitrectomy performed by a retina specialist to treat diseases of the posterior segment of the eye. This technique involves removing the vitreous gel to provide better access to the retina, allowing for repairs such as removing scar tissue, laser treatment of retinal detachments, and macular hole treatment. After the vitreous is removed, saline, a gas bubble, or silicone oil may be injected into the vitreous cavity to help hold the retina in position [16]. Anterior vitrectomy removes small portions of the vitreous humor from the front structures of the eye, often because the vitreous is tangled in an intraocular lens or other structures. This procedure may be done following eye trauma, during complex cataracts, cornea, or glaucoma surgery, or as a result of lens problems [17].

Interface vitrectomy involves using vitreous cutters, scissors, and forceps at the interface between silicone oil, air, or liquid perfluorocarbon (PFO) and the remaining vitreous, retinal, and retinal tissue. It is crucial not to apply a vacuum with the vitreous cutter or soft cannula within air, oil, or PFO bubbles to prevent blockage or air lock and minimize the risk of oil or PFO loss. Interface vitrectomy can help remove residual vitreous, while forceps membrane peeling is effective for managing epiretinal membranes, particularly in PVR [17]. Smaller gauge vitrectomy instruments, such as 23 gauge or 25 gauge, have become increasingly popular due to their advantages. These instruments allow for a sutureless, self-sealing sclerotomy, faster visual recovery, and reduced postoperative inflammation compared to traditional 20-gauge vitrectomy [18].

Retinal Detachment Repair

Scleral buckling and pneumatic retinopexy are two surgical techniques used to repair retinal detachment, a serious condition that can lead to vision loss if left untreated. Scleral buckling involves placing a silicone band or sponge (scleral buckle) around the sclera, the white part of the eye, to push the wall of the eye inward, relieving traction on the retina and allowing it to reattach. The buckle is secured under the conjunctiva and often permanently left in place. This procedure is often outpatient under local or general anesthesia, with success rates ranging from 80% to 90%. However, potential complications include infection, bleeding, increased nearsightedness, and recurrent retinal detachment [19]. Pneumatic retinopexy, in contrast, is a less invasive procedure involving the injection of a gas bubble into the eye to push the retina back into place. The retina is then sealed with laser photocoagulation or cryotherapy (freezing treatment). This procedure can often be performed in an ophthalmologist’s office. Pneumatic retinopexy is suitable for select cases with limited retinal detachment and specific retinal tear locations. The choice between scleral buckling and pneumatic retinopexy depends on factors such as the extent and location of the retinal detachment, the presence of vitreous traction, and the surgeon’s preference. Scleral buckling may be preferred for more complex detachments, while pneumatic retinopexy is suitable for select cases with specific characteristics [20].

Complications and Success Rates

PVR is a significant complication following surgery for RRD, occurring in approximately 5-10% of cases. Despite advancements in surgical techniques, PVR remains a challenging condition associated with less-than-optimal anatomical and visual outcomes. The overall primary success rate of PVR cases ranges between 43% and 69%. Following surgery for PVR-related detachments, the anatomical success rate has been reported to range from 45% to 85%. In cases presenting with established PVR, the primary overall success rate ranges from 43% to 69%. For severe PVR-related retinal detachments treated with primary scleral buckle surgery, the anatomical success rate ranges from 34% to 47% [15]. Final functional success rates following surgery for PVR-related detachments vary between 26% and 67%, with functional success typically defined in studies as achieving a final visual acuity of 5/200 or better. Despite multiple interventions, 10% to 40% of retinal detachments complicated by PVR cases remain detached despite repeated surgical attempts. In one study focusing on complex retinal detachments, predominantly involving PVR, only 26% of cases achieved a final visual acuity of 20/200 or better [1].

Pharmacological interventions

Anti-inflammatory Agents

Several anti-inflammatory agents have been explored as potential adjuncts to PVR surgical treatment. Corticosteroids, known for their broad anti-inflammatory and anti-proliferative effects, were among the first pharmacological interventions investigated. Promising preclinical studies demonstrated that a 2-mg dose of triamcinolone acetonide significantly reduced PVR-associated retinal detachment in a rabbit model, decreasing it from 90% to 56%. However, clinical trials have produced inconsistent results, with some studies showing no significant difference between treated patients and controls. Recent developments in sustained-release systems suggest they can maintain therapeutic drug concentrations over extended periods. However, a two-year randomized trial comparing dexamethasone implants to placebo in PVR patients found no significant differences in anatomical success or visual acuity outcomes [1]. While non-steroidal anti-inflammatory drugs (NSAIDs) have been investigated for their potential role in managing PVR, the available information is limited in demonstrating their efficacy compared to corticosteroids and other pharmacological agents, such as methotrexate (MTX) and anti-vascular endothelial growth factor (VEGF) therapies. Corticosteroids have shown promise in preclinical studies, but their effectiveness in clinical trials has been variable. Further research is necessary to establish their efficacy and safety profiles before they can be recommended as part of standard PVR management protocols. The current data does not provide sufficient evidence to conclude the efficacy of NSAIDs in treating PVR [21].

Anti-proliferative Agents

Several anti-proliferative agents have been studied for their potential in preventing and treating PVR, with 5-fluorouracil (5-FU) and MTX being prominent examples. 5-FU, a pyrimidine analog, inhibits DNA synthesis and cell division. Preclinical studies have shown that intravitreal injection of 5-FU can significantly reduce the occurrence and severity of PVR in animal models. Clinical trials have also assessed its efficacy when used adjunctively with surgery for PVR [22]. For instance, a randomized, double-blind trial investigated the combination of 5-FU and heparin in preventing PVR following retinal detachment surgery, finding a significant reduction in PVR incidence compared to a placebo. However, a more recent trial did not observe a significant difference in PVR rates between patients receiving 5-FU/heparin and those receiving a placebo [23]. MTX, another anti-proliferative agent, acts as a folate antagonist with anti-proliferative and anti-inflammatory properties. In vitro studies have demonstrated that MTX can inhibit the proliferation and migration of RPE cells and induce apoptosis, avoiding the photoreceptor toxicity associated with other agents such as 5-FU. Despite promising results in preclinical studies, the translation to clinical trials for MTX in PVR treatment has been less successful. Further research is necessary to establish its efficacy and safety profiles in human patients [24].

Anti-VEGF Therapy

Several studies have explored the potential of anti-VEGF therapies, particularly bevacizumab (Avastin), in treating PVR. Bevacizumab is a monoclonal antibody that binds to all isoforms of VEGF and has been used off-label for various ocular conditions. The rationale behind using bevacizumab for PVR stems from studies indicating elevated levels of VEGF in subretinal fluid and vitreous samples of PVR compared to uncomplicated retinal detachment [25]. A prospective, comparative, interventional study assessed the outcomes of intrasilicone bevacizumab injection after vitrectomy for severe PVR-related retinal detachment. The study found that this difference was not statistically significant, while retinal redetachment with PVR occurred in 47.3% of the bevacizumab-treated group and 36.8% of the control group. However, extensive subretinal fibrovascular proliferations were more prevalent in the bevacizumab group (55.5% vs. 14.3%) [26]. Another systematic in vitro study evaluated 36 pharmacological agents proposed for PVR treatment and identified dasatinib, resveratrol, simvastatin, and tranilast as promising candidates based on their anti-proliferative and anti-migratory effects on primary human RPE cells and cells derived from surgically excised human PVR membranes [27]. Ranibizumab is another anti-VEGF agent that inhibits biologically active forms of VEGF both in vitro and in vivo. However, specific information regarding the use of ranibizumab for PVR treatment was not found in the search results [28]. Further research is needed to clarify the efficacy and safety of anti-VEGF therapies such as bevacizumab and ranibizumab in managing PVR.

Emerging Drug Therapies

PVR complicates RRD by forming contractile membranes on the retinal surface, leading to tractional retinal detachment. Despite advancements in surgical techniques, managing PVR remains challenging, with less-than-optimal anatomical and visual outcomes [29]. Several therapeutic approaches have been investigated to prevent and treat PVR. Due to their anti-inflammatory and growth factor-reducing properties, corticosteroids have been explored as potential preventive therapies. However, clinical trials have produced varying and often disappointing results. Other pharmacological agents under active investigation include mitomycin C, bevacizumab, and ranibizumab. Mitomycin C applied intraocularly has shown promise in preventing PVR in severe traumatic retinal detachments, while intravitreal bevacizumab post-retinal reattachment surgery may benefit severe PVR cases. Ranibizumab inhibits multiple biologically active forms of VEGF in experimental models, yet clinical trials have not consistently shown benefits [2].

Adopting a more standardized surgical approach may facilitate the identification of novel adjunctive treatment options for PVR. Proper patient selection and standardized surgical techniques are critical for evaluating the efficacy of pharmacological interventions. Various agents, including corticosteroids, anti-proliferative, and anti-neoplastic agents, have shown potential in reducing postoperative PVR risk, yet none have been approved as adjuncts to surgical treatment [15]. Emerging drug therapies such as tyrosine kinase inhibitors (dasatinib), antioxidants (resveratrol), statins (simvastatin), and anti-inflammatory agents (tranilast) are being investigated for their anti-proliferative and anti-migratory effects on human retinal cells derived from PVR membranes in systematic in vitro studies. Peri-surgical pharmacological interventions are promising adjuvant therapies to reduce PVR and improve surgical outcomes. Developing sustained dual drug delivery systems may enhance the effectiveness and precision of drug delivery within the vitreous cavity [27]. While these emerging therapies show potential, further research, including in vivo studies and clinical trials, is essential to establish their safety and efficacy in treating PVR. Innovations in drug delivery systems may also enhance the bioavailability and efficacy of these agents. Currently, no approved medical therapy exists for the treatment or prevention of PVR, highlighting the need for continued research to advance our understanding and develop effective therapeutic strategies for improving outcomes in affected patients [30].

Biological and Genetic Therapies

Mesenchymal stem cells (MSCs) and endothelial progenitor cells (EPCs) have shown promise in preclinical studies for treating PVR. MSCs can differentiate into various cell types and secrete growth factors aiding tissue repair. Similarly, EPCs are studied for their potential to enhance angiogenesis and improve retinal function in animal models of PVR. However, clinical trials assessing stem cell therapies for PVR remain limited. Further research is essential to determine the optimal cell type, delivery method, and timing of administration to maximize therapeutic efficacy [31]. Bioinformatics tools are used for gene enrichment analysis to identify genes involved in PVR pathogenesis and potential drugs interacting with these genes. Drug repurposing is another strategy aimed at identifying existing drugs that could be repurposed for PVR treatment based on their interactions with PVR-related genes. Promising repurposed drugs identified through this approach include curcumin, which has anti-inflammatory and antioxidant properties, statins known for cardiovascular benefits and potential in PVR prevention, and prednisone and MTX, corticosteroid and anti-neoplastic agents currently under investigation in clinical trials for PVR [30]. Connective tissue growth factor plays a crucial role in fibrosis regulation and is considered a potential therapeutic target for PVR based on in vitro studies. Anti-VEGF agents such as bevacizumab and ranibizumab have been explored for their potential benefits in severe PVR when administered via intravitreal injection after retinal reattachment surgery. Despite these advancements, no biological or genetic therapies have been approved to treat PVR. Validation through further preclinical and clinical studies is necessary to establish the efficacy and safety of these novel therapeutic approaches [32].

Novel and Experimental Treatments

Using biomaterials and scaffolds represents a promising approach for treating PVR. Biomaterial scaffolds carry cells, drugs, and growth factors, creating a stable microenvironment that supports tissue regeneration. Polymeric biomaterials can be engineered to act as endotamponade agents, preventing intraocular scarring in cases of retinal detachment. Biodegradable scaffolds offer advantages such as adjustable mechanical properties, customizable inner structures, and the ability to degrade over time, which are particularly beneficial for PVR treatment [33]. Combining biomaterial scaffolds with hydrogels enhances their therapeutic effects. For instance, nanostructured microguidance scaffolds made from polycaprolactone and poly(lactic-co-glycolic acid) (PLGA), synthesized through electrospinning and implanted with self-assembled RADA16-I-BMHP1 hydrogels, have been shown to promote axon regeneration, myelination, and motor function recovery in chronic spinal cord injury (SCI) models [34]. Similarly, biodegradable scaffolds designed to deliver drugs or growth factors in a controlled manner, with different layers releasing substances at various stages of PVR treatment, show promise. Examples include PLGA scaffolds loaded with recombinant human neurotrophin-3, which have demonstrated axonal regeneration and functional recovery in animal models of SCI [34]. Nanotechnology-based drug delivery systems using biomaterial scaffolds are also under investigation for PVR treatment. These systems allow for the localized release of growth factors or neuroprotective agents in injectable gel forms. Concepts developed for SCI treatments using degradable and non-degradable biomaterial scaffolds highlight potential applications for PVR therapy [35].

Multimodal and combined therapies

Synergistic Effects of Combined Approaches

Synergistic effects, where the combined effect of two or more therapies exceeds the sum of their individual effects, offer significant potential in treating complex diseases such as cancer and PVR. This approach is appealing as it can enhance therapeutic efficacy while potentially reducing toxicity. Combining therapies may outperform single-agent treatments by simultaneously targeting multiple pathways or mechanisms, particularly in diseases with intricate pathophysiology [36]. Numerous studies across different disease contexts have demonstrated synergistic effects of combination therapies. For instance, in lung cancer, combining piperlongumine, a natural compound, with gemcitabine, a chemotherapeutic agent, showed synergistic inhibition of KRAS mutant cells. Another example involves hemin, which disrupts TIGIT-PVR interactions, inducing ferroptosis and enhancing cancer immunotherapy efficacy. Similarly, in canine models of mitral valve disease, combining beraprost sodium and sildenafil resulted in synergistic reductions in pulmonary vascular resistance and left-heart loading [37]. Mechanistically, synergistic effects can arise from multi-target interactions, where combined therapies act on multiple pathways concurrently, thereby amplifying their effectiveness. Cooperative activity, where therapies complement each other in specific cellular contexts or disease states, also contributes to synergistic effects. Furthermore, combination therapies may overcome resistance mechanisms that develop against single-agent treatments [38]. Despite their promise, synergistic combination therapies pose challenges. Optimizing the combination and determining the ideal dosing can be complex and require extensive testing. Careful monitoring is crucial, as synergistic combinations can increase the risk of toxicity if not properly managed. Moreover, the intricate nature of biological systems and the interactions between different pathways make predicting and controlling synergistic effects challenging [39].

Case Studies and Clinical Outcomes

Case studies and clinical outcomes are pivotal in assessing the efficacy and safety of novel therapeutic strategies for PVR. Case studies offer valuable real-world insights into applying new therapies, highlighting potential benefits, risks, and challenges. For instance, case reports have documented the use of intravitreal aflibercept injections in combination with surgery, leading to improved anatomical and visual outcomes in PVR patients. Similarly, gene therapy approaches targeting key molecules implicated in PVR development have shown promising results in individual cases [15]. Clinical outcomes from larger trials are essential for evaluating the broader efficacy and safety of new PVR therapies. Clinical trials have investigated slow-release dexamethasone implants alongside standard surgical treatments, demonstrating superior effectiveness in reducing PVR recurrence compared to standard treatments alone. Studies evaluating intravitreal 5-FU and heparin as adjunctive therapies have also reported enhanced anatomical and visual outcomes in treated groups versus controls [40]. Despite these advancements, several challenges and limitations exist in interpreting findings from case studies and clinical trials in PVR. Many studies have small sample sizes, limiting the generalizability of results to broader patient populations. PVR itself is heterogeneous, varying in severity and associated risk factors, complicating direct comparisons across studies. Moreover, the long-term consequences of PVR necessitate studies with extended follow-up periods to fully assess the sustained impact of new therapies [41].

Personalized Medicine in PVR Treatment

Personalized medicine offers a promising avenue for improving treatment outcomes in PVR by tailoring therapies to individual patient characteristics. PVR is a multifaceted disease influenced by both genetic predispositions and environmental factors. Personalized medicine approaches can be pivotal in identifying high-risk patients and guiding targeted preventive and therapeutic interventions [42]. Genetic factors significantly contribute to PVR susceptibility, as evidenced by genome-wide association studies identifying relevant genetic variants. These variants often involve genes associated with inflammation, cell proliferation, and extracellular matrix remodeling, providing insights into individualized risk assessment for PVR. Stratifying patients based on their genetic profiles enables more vigilant monitoring and proactive treatment strategies [43]. Pharmacogenomics, which explores how genetic variations affect medication responses, is crucial in optimizing PVR treatments. It helps select the most effective therapies and determine appropriate dosages, thereby minimizing the need for trial-and-error approaches and potentially reducing treatment-related adverse effects. This tailored approach enhances treatment efficacy for individual patients [44]. Precision diagnostics, including genetic and molecular biomarkers, are pivotal in early PVR detection and treatment decision-making. Biomarkers, such as specific cytokine levels in vitreous or aqueous humor, can provide insights into disease progression and guide the intensity of surgical interventions or adjunct therapies. Early identification through precision diagnostics improves the chances of successful treatment outcomes [44]. Despite these advancements, challenges remain in translating personalized medicine approaches into clinical practice for PVR. Large-scale validation studies are needed to confirm the utility of genetic and molecular biomarkers in diverse patient populations. Additionally, developing cost-effective and scalable diagnostic platforms is essential for widespread implementation. Integrating personalized medicine strategies into clinical workflows requires careful consideration of logistical and regulatory aspects to ensure effective patient management [44].

Challenges and Future Directions

**Figure 1:**
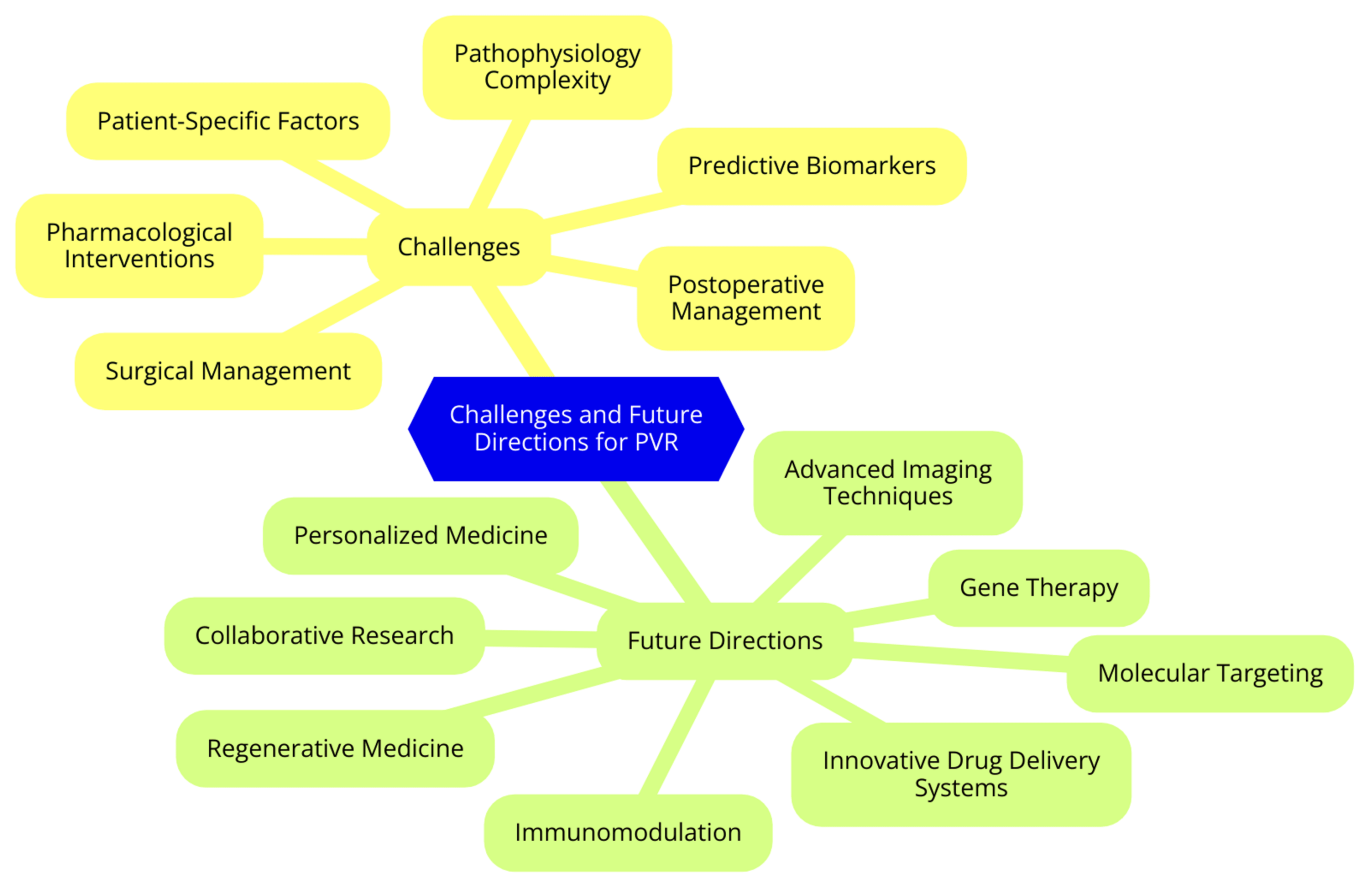
Challenges and future directions for proliferative vitreoretinopathy (PVR). Image credit: Dr. Kasturi K. Dhawale.

PVR continues to pose a significant challenge in managing RRD despite advancements in surgical techniques and adjunctive therapies. Current treatments, including longer-acting gases and long-term vitreous substitutes such as silicone oil, achieve 60-75% success rates at six months postoperatively. However, over one-fourth of initially successful cases experience re-detachment due to recurrent retinal traction, with PVR being a major contributing factor [45]. In addressing these challenges, techniques such as extraskeletal cryo coagulation for localized intraretinal PVR have shown promise by preventing further proliferation and reducing the risk of re-detachment caused by traction. However, concerns remain about potential long-term subretinal cell migration that could lead to recurrent PVR and subsequent re-detachment [46]. Advancements in wide-angle viewing systems, advanced vitrectomy machines, and small-gauge instruments have significantly improved surgical outcomes for PVR. Techniques such as perfluorocarbon liquid, high-speed cutters, and long-term heavy silicone oil tamponades have improved anatomical results and reduced recurrence rates [47]. Current research efforts are focused on deepening our understanding of the complex pathophysiology of PVR and developing novel therapeutic strategies. This includes targeting specific signaling pathways, such as transforming growth factor-beta and platelet-derived growth factor, crucial in PVR development. Gene therapy approaches to modulate the expression of key molecules involved in PVR pathogenesis are also being explored. Despite promising preclinical results, translating these findings into successful clinical applications remains a significant hurdle, necessitating further rigorous research to establish efficacy and safety [48]. Challenges and future directions for PVR are shown in Figure 1.

## Conclusions

PVR remains a significant challenge in ophthalmology, particularly due to its potential to cause vision loss following retinal detachment surgery. Recent advancements in surgical techniques, pharmacological interventions, and biological therapies have marked significant progress in preventing and treating PVR. Innovations such as improved vitrectomy techniques, anti-inflammatory and anti-proliferative drugs, and the exploration of gene and stem cell therapies have opened new avenues for managing this complex condition. Despite these advancements, the recurrence of PVR and the long-term patient outcomes continue to pose challenges, underscoring the need for ongoing research and development. Future efforts must focus on early detection, personalized medicine approaches, and integrating multimodal therapies to enhance treatment efficacy and patient outcomes. By continuing to advance our understanding and treatment of PVR, we can work toward reducing its burden and improving the quality of life for affected individuals.
